# Prognostic factors in children and adolescents with differentiated thyroid carcinoma treated with total thyroidectomy and RAI: a real-life multicentric study

**DOI:** 10.1007/s00259-021-05586-8

**Published:** 2021-10-19

**Authors:** Angelina Cistaro, Natale Quartuccio, Maria Carmen Garganese, Maria Felicia Villani, Claudio Altini, Milena Pizzoferro, Arnoldo Piccardo, Manlio Cabria, Michela Massollo, Mohamad Maghnie, Alfredo Campennì, Massimiliano Siracusa, Sergio Baldari, Stefano Panareo, Luca Urso, Mirco Bartolomei, Diego De Palma, Armando Grossi, Angelica Mazzoletti, Francesco Dondi, Francesco Bertagna, Raffaele Giubbini, Domenico Albano

**Affiliations:** 1 Associazione Italiana Medicina Nucleare (AIMN), Pediatric Study Group, Milan, Italy; 2 Nuclear Medicine Division, Salus Alliance Medical, Genoa, Italy; 3 Medicine Unit, A.R.N.A.S. Ospedali Civico, Di Cristina E Benfratelli, Palermo, Italy; 4 Imaging Department, Nuclear Medicine Unit, IRCCS Bambino Gesù Pediatric Hospital, Piazza Sant’Onofrio 4, 00165 Rome, Italy; 5 Department of Nuclear Medicine, E.O. “Ospedali Galliera”, Mura delle Cappuccine 14, 16128 Genoa, Italy; 6 Department of Pediatrics, IRCCS Istituto Giannina Gaslini, Genova, Italy; 7 Department of Biomedical and Dental Sciences and Morphofunctional Imaging, University of Messina, Messina, Italy; 8 Nuclear Medicine Unit, University Hospital “G. Martino”, Messina, Italy; 9 Nuclear Medicine Department, Azienda Ospedaliera Universitaria Di Modena, Modena, Italy; 10 Nuclear Medicine Unit, Oncological Medical and Specialist Department, University Hospital of Ferrara, Ferrara, Italy; 11 Department of Nuclear Medicine, ASST Sette Laghi, Ospedale di Circolo e Fondazione Macchi, Varese, Italy; 12 Endocrine Pathology of Chronic and Post Cancer Diseases Unit, IRCCS Bambino Gesù Pediatric Hospital, Piazza Sant’Onofrio 4, 00165 Rome, Italy; 13 Nuclear Medicine Department, University of Brescia and ASST Spedali Civili Di Brescia, P.le Spedali Civili 1, 25123 Brescia, Italy

**Keywords:** Differentiated thyroid carcinoma, Pediatric, Thyroglobulin, RAIT, Prognosis, ATA

## Abstract

**Purpose:**

This multicentric study aimed to investigate the main prognostic factors associated with treatment response at 1 year after radioactive iodine therapy (RAIT) and the last disease status in pediatric patients affected by differentiated thyroid carcinoma (DTC).

**Materials and methods:**

In the period 1990–2020, all consecutive patients ≤ 18 years from six different centers were retrospectively included. Patients were classified as low, intermediate, and high risk for persistence/recurrence. The response to RAIT was evaluated and scored 1 year later according to 2015 ATA guidelines. Moreover, at the last follow-up, the disease status was evaluated and dichotomized as no evidence of disease (NED) or persistent disease.

**Results:**

Two hundred and eighty-five patients (197 female, 88 male; mean age 14.4 years) were recruited. All, except nine, underwent near-total thyroidectomy followed by RAIT. One-year after first RAIT, 146/276 (53%) patients had excellent response, 37/276 (14%) indeterminate response, and 91/276 (33%) incomplete response. One-year after RAIT, children with excellent response had significantly lower stimulated thyroglobulin (sTg) compared to not excellent group (median sTg 4.4 ng/ml vs 52.5 ng/ml, *p* < 0.001). ROC curve showed sTg higher than 27.2 ng/ml as the most accurate to predict 1-year treatment response. After a median follow-up of 133 months, NED was present in 241 cases (87%) while persistent disease in 35 (13%). At multivariate analysis, sTg and 1-year treatment response categories were both significantly associated with the last disease status (*p* value 0.023 and < 0.001).

**Conclusions:**

In pediatric DTC, sTg is significantly associated with 1-year treatment response and final outcome. However, 1-year response is the principal prognostic factor able to predict pediatric DTCs outcome.

**Supplementary Information:**

The online version contains supplementary material available at 10.1007/s00259-021-05586-8.

## Introduction

Although differentiated thyroid carcinoma (DTC) is the most frequent endocrine pediatric carcinoma, it is a rare disease in childhood and among adolescents [1]. Its incidence increases over the entire adolescence and particularly among females [2]. At the onset DTC, pediatric patients have higher risk of cervical lymph node and lung metastases, when compared to the adult counterpart [3]. However, the prognosis is favorable, and the mortality rate is very low [4–6]. In this particular setting, customizing the risk of relapse of each patient may avoid unbeneficial treatment.

For the first time in 2015, American Thyroid Association (ATA) published specific guidelines for pediatric patients affected by DTC [7]. These guidelines introduced a new classification, based on post-surgical features (i.e., TNM), which divides DTCs in patients at low, intermediate, and high risk of relapse. The goal of this stratification system was not strictly to define the risk of disease mortality but rather to identify those patients at higher risk of disease persistent/recurrence with the aim to recognize who should undergo further treatments. However, this risk classification derived directly from the adults’ guidelines [3] has never been extensively validated in large pediatric population.

The initial treatment consists of total thyroidectomy followed by postoperative radioactive iodine therapy (RAIT) whenever indicated. The effective role and benefit of RAIT has never been fully clarified, especially in children at low and intermediate risk, whereas a significant benefit in terms of recurrence rate and overall survival was reported in DTC patients at highest risk [8]. The goal would be to maintain the low disease-specific mortality currently experienced by children with DTC as well as to reduce the potential complications of therapy and over-treatment [9].

Since an increasing incidence of the DTC has been observed [10], the detection of factors predicting treatment response and survival is of clinical interest.

In adults, the pre-ablation stimulated thyroglobulin (sTg) had high predictive value in identifying the risk of disease persistence/recurrence after initial treatment and overall survival [3, 8, 11] regardless the DTC stage. Instead, the role of sTg in DTC pediatric patients remains to be fully established [12–15]. At the same time, the usefulness of ATA risk classification in children with DTC has been evaluated in few studies [16–21], and also the 1-year response to initial therapy categories presented in the 2015 adults’ guideline (i.e., excellent response, biochemical incomplete response, structural incomplete response and indeterminate response) [3] has been tested few cases of pediatric population.

In this clinical scenario, the aim of this study was to investigate the role of some risk factors, such as sTg and ATA risk classification, in predicting treatment response at 1 year after RAIT.

The second aim was to investigate whether the same variables and the dynamic risk classification proposed 1 year after RAIT derived by adults ATA guidelines [3] may predict the last disease status.

## Materials and methods

### Patients

The DTC databases of six Italian Nuclear Medicine Departments were retrospectively screened to retrieve all DTC pediatric patients (age 0–18) treated between 1990 and 2020**.**

Inclusion criteria were histological diagnosis of DTC; age less or equal to 18 years at the time of diagnosis; surgery (thyroidectomy plus lymph-node dissection, if necessary) and RAIT performed; and at least 12 months of follow-up after RAIT.

Exclusion criteria were more than 18 years old; the absence of at least 12 months of follow-up; and the lack of RAIT performed. All selected DTCs were reclassified according to the latest TNM version [22].

About the exclusion criteria, we decided to include also patients with previous radiotherapy or chemotherapy treatments (so with a previous cancer) to evaluate the potential predictive and prognostic role of this feature.

All patients were admitted to our Nuclear Medicine Departments for the ablation of thyroid remnant and any subsequent radiometabolic therapies if needed, according to AIMN (Italian Association of Nuclear Medicine), EANM (European Association of Nuclear Medicine), and ATA guidelines differently applied according to the version in force during the patient’s treatment period [3, 7, 22]. The administered activity of RAI was established according to the risk class based on the TNM staging of the American Joint Committee on Cancer/International Union against Cancer currently in use, the status of the disease and on a scintigraphic evaluation with an empirical approach. In selected cases, scintigraphy was implemented by dosimetry (when feasible and available) and RAI administered estimated with a dosimetric approach.

The main epidemiological features (gender, age at diagnosis, puberty status, familiarity for DTC, previous oncological diseases, and related therapies), post-surgical histopathological data (i.e., tumor histology, tumor size, capsular invasion, vascular invasion, presence of Hashimoto thyroiditis, multicentricity, lymph node, and distant metastases involvement), biochemical data at the time of the first RAIT (TSH, Tg, anti-Tg antibody measurements), ATA class risk (low, intermediate, or high) [7], and information related to the RAI administration (first RAI activity administrated, total amount of RAI administered, total number of radiometabolic therapies) were collected.

This study was approved by the ethics committee of ASST Spedali Civili di Brescia Hospital (NP 4258) as principal investigator and by ethics committee of other hospitals.

### Laboratory analysis

Serum Tg was measured using immunoradiometric assays (radioimmunoassay, electrochemiluminescence, chemiluminescence-ECLIA) according to each local institute protocol and expressed as ng/ml. Normal ranges were 0–40 ng/ml (radioimmunoassay), < 55 ng/ml (electrochemiluminescence), and 3.5–77 ng/ml (ECLIA), respectively. Tg antibodies (TgAb) were measured using the passive agglutination method and chemiluminescence and dichotomized in positive (for Tg interference) or negative (not interference) according to each institutional normality range. TgAb was dichotomized in positive and negative according to the normal values of the local laboratory measurement. TSH levels were measured by electrochemiluminescence immunoassay and expressed in mIU/l.

### Follow-up and outcomes

The median follow-up time was 133 ± 100 months (range 12–360 months). Patients were followed every 6–12 months with physical examinations and laboratory measurements (Tg on levothyroxine therapy or sTg in selected cases, TgAb, and TSH level) and with several imaging procedures, such as neck ultrasound, RAI diagnostic whole body scan (DxWBS), or other as appropriate. The disease status was registered and updated at each evaluation. DxWBS was performed after the administration of 370 MBq of RAI about 12 months after the first radiometabolic therapy in all cases, except of patients that received a new RAIT for persistence of disease or incomplete response.

The clinical status of each patient one-year after the first RAIT has been re-classified according to the therapy categories classification of 2015 ATA guidelines [3], which identifies four classes of response (excellent response, biochemical incomplete response, structural incomplete response, and indeterminate response) based on a combination of imaging (RAI WBS, neck ultrasound, and any additional imaging exams) and biochemical (TSH, Tg, and TgAb) findings.

Any additional therapies (surgery or further RAIT) were decided based on either radiological evidence of persistent/progressive disease (structural disease), evidence of persistent positive Tg, or rising Tg/TgAb (biochemical disease) in compliance with the guidelines in force at that time.

Furthermore, at the last control, the patients were classified as having no evidence of disease (NED) or with persistent disease, based on a combination of laboratory and imaging features. NED was defined by the absence of cervical lymph node metastases or local relapse on a recent neck ultrasound, no evidence of structural disease and either suppressed Tg < 1 ng/ml or sTg < 2 ng/ml (without the presence of TgAb) [23]. In case of positive TgAb, NED was considered if declining anti-Tg antibody and concomitant negative neck ultrasound and/or negative DxWBS [23]. Instead, pediatric patients who did not fulfill the previous mentioned criteria for NED at the last follow-up were defined as having persistent disease.

### Statistical analysis

Statistical analysis was carried out using Statistical Package for Social Science (SPSS) version 23.0 for Windows (IBM, Chicago, Illinois, USA) and MedCalc Software version 18.1 (Ostend, Belgium). The descriptive analysis of categorical variables was summarized by the calculation of simple and relative frequencies while the continuous variables by median, mean, standard deviation, and range values. The statistical significance of the continuous variables was tested with a Student’s *t*-test or Mann–Whitney’s *U*-test, and a χ2 test was performed for the categorical variables. A *p* value of ≤ 0.05 was considered statistically significant. The Youden index from the receiver operating characteristic (ROC) curve was applied as a criterion for selecting the best threshold point for sTg to predict 1-year treatment response (excellent response vs not excellent response) and this result was compared with several sTg thresholds (2, 5, and 10 ng/ml) suggested in ATA guidelines [7]. Hazard ratios with 95% confidence intervals (CI) were calculated by univariate and multivariate Cox regression analysis.

For the evaluation of the last disease status, only patients with more than 3 years of follow-up were included.

Univariate logistic regression analysis was applied to evaluate the ability of the main clinicopathological factors to predict long-term outcome (NED vs persistence of disease). Multivariate regression analysis was applied to evaluate the ability of the aforementioned features. A two-tailed *p* < 0.05 was considered statistically significant.

## Results

### Patient selection

Totally 303 pediatric patients with a histological diagnosis of DTC were initially recruited in the study. Eighteen patients were lost immediately after the diagnosis and were excluded by the analysis. All the remaining 285 patients underwent a near-total thyroidectomy, associated with central neck dissection in 170 cases and lateral neck dissection in 102 cases.

All patients underwent total thyroidectomy; all operations were performed by local experienced thyroid surgeons (all with more than 10 years of experience in thyroid surgeries). Prophylactic central lymph node dissection was not routinely performed, but only in case of suspected or pathologically confirmed N1a disease or when advanced primary tumors (T3 or T4) were noted. Lateral compartment neck dissection was performed in case of clinically suspicious or pathologically (based on imaging and/or cytology) confirmed N1b disease.

After surgery, 276 out of 285 (97%) patients received RAIT (eight patients affected by papillary microcarcinoma and one died few days after thyroidectomy for surgical complications did not) (Supplemental Fig. 1).

**Fig. 1:**
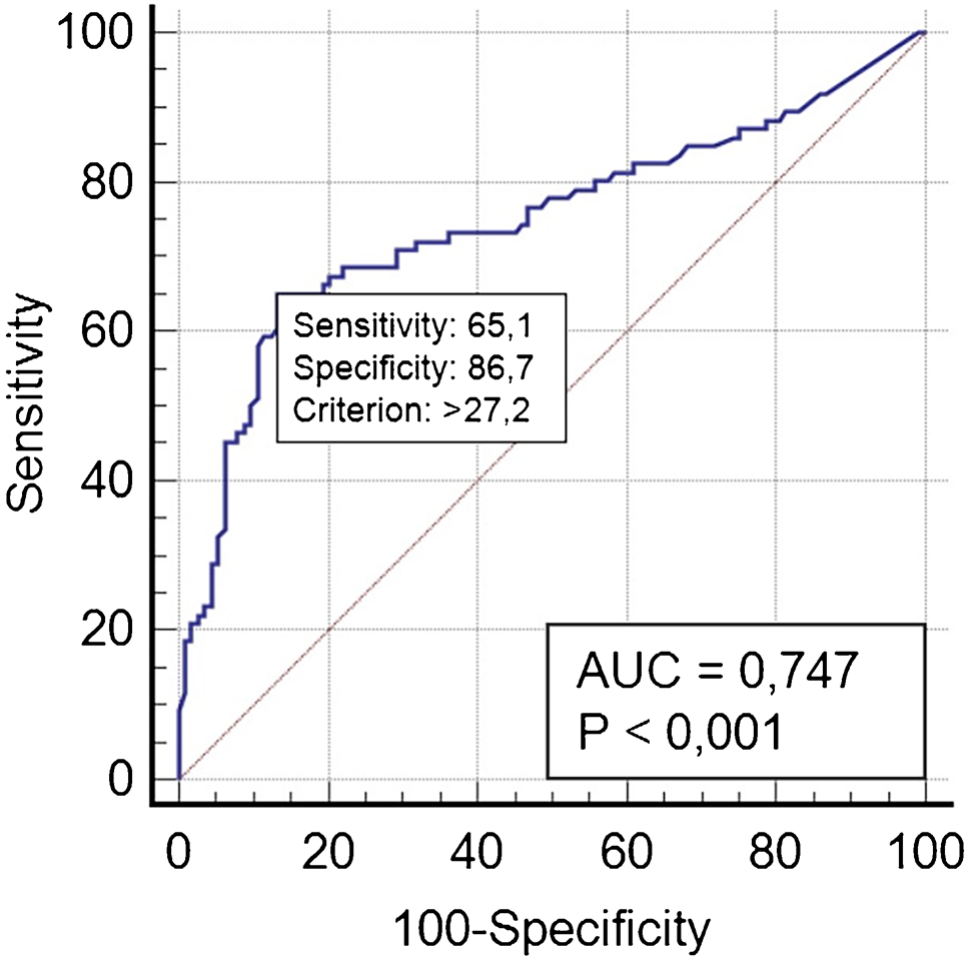
ROC curve analysis which evaluated the role of stimulated Tg in ng/ml for predicting treatment response 1 year after RAIT

A second operation was performed only in six cases when local relapse or nodal disease was discovered.

Before the first RAI administration, levothyroxine was discontinued for 20–30 days in two hundred and forty-seven patients, while in the remaining 29 patients, recombinant human thyrotropin (rhTSH) (Genzyme Corporation) was administered intramuscularly with a dose of 0.9 mg on 2 consecutive days during treatment with levothyroxine; in these patients, RAIT was administered the day after the second rh-TSH injection. rh-TSH injection was an off-label use authorized by local center, and a specific informed consent was signed by each patient and previously investigated by other authors in the diagnostic field [24].

### Patient features

The patients’ age ranged from 4 to 18 years with a median age of 15; there was a prevalence of female (F:M = 2.2:1).

In most cases, the diagnosis of DTC was done after 2010 (n 120), with a prevalence higher than the previous 2 decades (1990–2002 and 2001–2010).

All pediatrics had a histopathological diagnosis of DTC: 154 classic variant of papillary carcinoma, 59 follicular variant of papillary carcinoma, 41 aggressive papillary variants (17 tall cells variant, 11 diffuse sclerosing variant, 2 columnar-cell variant, 7 solid variant and 1 hobnail variant of papillary carcinoma, and 3 poorly differentiated carcinoma), 18 follicular carcinoma (10 were minimally invasive and 8 widely invasive), 3 Hurtle cell carcinoma, and one case of noninvasive follicular thyroid neoplasm with papillary-like nuclear features (NIFPT).

Tumor size of primary carcinoma was 22 ± 14 mm (range 9–90 mm). Multicentricity of neoplastic lesions was present in 100 cases (37%), capsular invasion in 138 (50%), and vascular invasion in 89 (32%).

The median administered activity of first RAIT was 2.7 GBq (interquartile 1–3.7), while the median cumulative RAI activity administered per patient was 3.7 GBq (interquartile 1.1–37). RAI activities administered depended on several factors such as surgery extent, tumor size, presence of metastases, pubertal stage, and body weight. Often, the choice of activity had been discussed in the local multidisciplinary tumor board groups. For children in pre-puberty or post-puberty with low-risk disease, the activity administered was usually 1.1 GBq; in case or post-puberty with intermediate-high risk, the median activity was 2.5 GBq with a maximum of 3.7 GBq.

Thirty-four out of 276 patients had a previous history of oncological disease: chronic lymphatic leukemia (CLL) in 19 cases, Hodgkin lymphoma in 4, medulloblastoma in 2, neuroblastoma in 2, non-Hodgkin lymphoma in 1, breast cancer in 1, Wilms kidney tumor in 1, astrocytoma in 1, acute myeloid leukemia in 1, dysgerminoma in 1, and a concomitant breast and clear cell renal carcinoma in 1. Among them, 32 underwent radiotherapy and/or chemotherapy and two surgical interventions. In patients without TgAb interference (*n* = 203), sTg at the time of first radioiodine treatment was 92 ± 482 ng/ml (range 0.04–5940); TgAb were present in 73 patients (26%). The patient’s features are summarized in Table 1.

**Table 1:** Baseline features of our 276 patients

	Average ± SD (range)	Median	Patients *n* (%)
Age years	14.4 ± 3.1 (4–18)	15	
Pre puberty			64 (23%)
During-after puberty			212 (77%)
Gender			
Male			85 (31%)
Female			191 (69%)
Familiarity for DTC			42 (15%)
Thyroidectomy total			276 (100%)
Lymphadenectomy central			170 (62%)
Lymphadenectomy lateral			102 (37%)
Decade of diagnosis of DTC			
1990–2000			68 (25%)
2000–2010			88 (32%)
2011–2020			120 (43)
Histotype			
Papillary			154 (58%)
Follicular variant of papillary			59 (22%)
Follicular			18 (6.6%)
Aggressive variant			41 (15%)
Hurtle cell			3 (1.1%)
NIFTP			1 (0.4%)
Capsule invasion			138 (50%)
Vascular invasion			89 (32%)
Tumor size (mm)	22 ± 14 (9–90)	20	
Multicentricity			100 (37%)
Hashimoto thyroiditis			64 (24%)
T-stage			
sT1a			40 (14%)
sT1b			61 (22%)
sT2			80 (29%)
sT3			74 (27%)
sT4			19 (7%)
sTx			2 (1%)
N-stage			
sN0			105 (38%)
sN1a			90 (33%)
sN1b			81 (29%)
M-stage			
sM0			236 (85%)
sM1			40 (15%)
Stage			
sN1M1			26 (9%)
ATA class risk			
Low			84 (30%)
Intermediate			141 (51%)
High			51 (19%)
Previous RTT and/or CMT			32 (11%)
Previous oncological disease			34 (12%)
I131 therapy			276 (100%)
TSH stimulation			
Withdrawal			247 (90%)
rhTSH			29 (10%)
sTg at the time of ablation (ng/ml)	92 ± 482 (0.04–5940)	5.55	
TgAb positive at ablation			73/276 (26%)
First RAI activities administrated (GBq)	2.6 ± 1.25 (1–7.4)	2.7	
Cumulative RAI activities administrated (GBq)	9.6 ± 15.2 (1–53)	3.7	
N° radiometabolic therapies	2 ± 1.9 (1–10)	1	
1-year treatment response categories			
Excellent response			146 (53%)
Indeterminate response			37 (13%)
Biochemical and/or structural incomplete response			91 (33%)
na			2 (1%)
Last disease status			
NED			241/276 (87%)
FU time months	133 ± 100 (1–360)	110	

*SD* standard deviation, *n* number, *GBq* Gigabequerel, *var* variant, *sTg* stimulated thyroglobulin, *Ab* antibodies, *na* not available, *NIFTP* noninvasive follicular thyroid neoplasm with papillary-like nuclear features, *RTT* radiotherapy, *CHT* chemotherapy, *RAI* radioactive iodine, *NED* not evidence of disease, *FU* follow-up

### Treatment response after 1 year

At 1 year after the first RAIT, 146 (53%) children showed excellent response, 37 (14%) indeterminate response, and 91 (33%) incomplete response. The remaining 2 patients were lost during the follow-up before the first year (Table 1). Among incomplete response, 79 had both biochemical and structural incomplete response, 7 only biochemical incomplete response, and 4 only structural incomplete response.

**Fig. 2:**
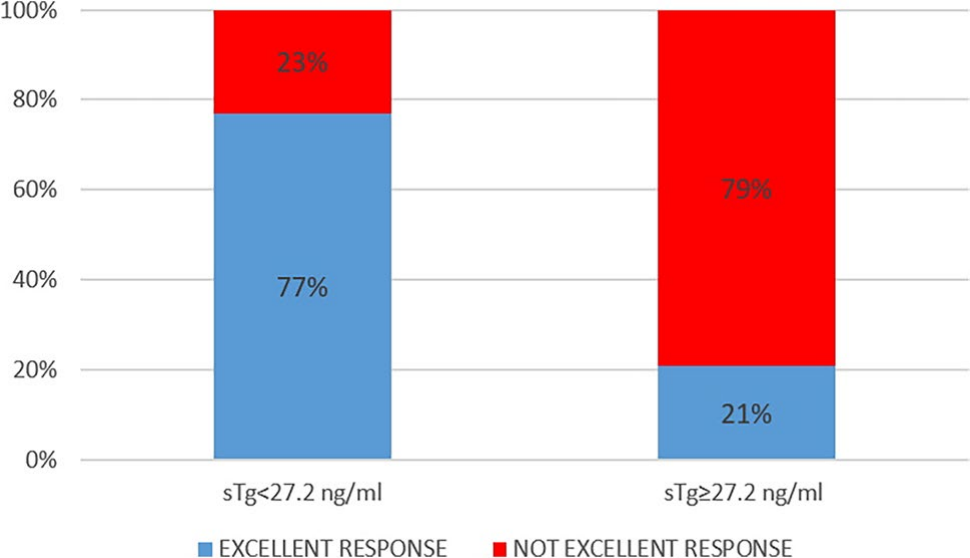
Comparison between 1-year response rate in patients with high or low baseline stimulated Tg after 1 year

At univariate analysis, children with excellent response at 1 year after RAIT were significantly younger (*p* = 0.043), had less frequently capsular (*p* < 0.001) and vascular invasion (*p* < 0.001), had smaller tumor size (*p* = 0.007) and sTg (*p* < 0.001) and less frequently presence of TgAb (*p* = 0.036), and presented more frequently ATA low-risk disease (*p* < 0.001) compared to not excellent response group (including indeterminate response and incomplete response) (Table 2). Only sTg confirmed to be an independent predictor of treatment response at multivariate analysis (HR 5.02, *p* < 0.001). However, also baseline anti-Tg antibodies confirmed to be more frequent in not excellent response group than excellent response group (HR 2.22, *p* = 0.040). ROC analysis showed that a pre-ablation sTg ≥ 27.2 ng/ml was the best threshold to discriminate excellent response from not excellent response with a sensitivity of 65.1% (95%CI 54.1–75.1), specificity 86.7% (95%CI 79.1–92.4), and AUC of 0.747 (Fig. 1). In fact, the 81 patients with a sTg ≥ 27.2 ng/ml showed a higher rate of not excellent response 1 year after RAIT (79%) as compared with those 182 patients with sTg level < 27.2 ng/ml (not excellent response rate 23%) (Fig. 2).

**Table 2:** Comparison between DTC patients with excellent response and not excellent response 1 year after RAIT

Variable			Univariate	Multivariate	
	Excellent response *n* 146	Not excellent response *n* 128	*p* value	HR (95%CI)	*p* value
Age, mean (years)	14.8	14	0.043	1.022 (0.926–1.127)	0.663
Gender F:M	105:41 (72%:28%)	85:43 (66%:34%)	0.171		
Puberty	118 (81%)	93 (72%)	0.109		
Decade of diagnosis			0.425		
1990–2000 + 2001–2010	79 (54%)	75 (58%)			
2011–2020	67 (46%)	53 (42%)			
Histotype			0.120		
Papillary	80 (55%)	72 (56%)			
Follicular var of papillary	30 (21%)	29 (23%)			
Follicular	12 (7.4%)	6 (5%)			
Aggressive variant	20 (14%)	21 (16%)			
Hurtle cell	3 (2%)	0			
NIFPT	1 (0.6%)	0			
Capsule invasion	56 (38%)	81 (63%)	< 0.001	1.602 (0.849–3.027)	0.145
Vascular invasion	32 (22%)	57 (44%)	< 0.001	1.236 (0.620–2.464)	0.547
Thyroiditis	29 (14%)	35 (27%)	0.154		
Tumor size, mean (mm)	19	25	0.007	1.157 (0.935–1.431)	0.179
Multicentricity	48 (33%)	51 (40%)	0.249		
ATA class risk			< 0.001	1.390 (0.845–2.286)	0.194
Low	77 (46%)	24 (19%)			
Intermediate	79 (47%)	65 (51%)			
High	12 (7%)	39 (30%)			
History of cancer	17 (12%)	16 (12.5%)	0.739		
sTg at the time of ablation, mean (ng/ml)	19.8	126.1	< 0.001	5.02 (2.440–10.330)	< 0.001
TgAb positive at ablation	31 (21%)	42 (32%)	0.036	2.22 (1.154–3.345)	0.040
First RAI activities administrated, mean (GBq)	2.7	2.7	0.680		

*F* female, *M* male, *RAIT* radioactive iodine therapy, *NIFPT* noninvasive follicular thyroid neoplasm with papillary-like nuclear features, *sTg* stimulated thyroglobulin, *Ab* antibodies

Investigating the principal sTg thresholds reported in literature (such as 10 and 2 ng/ml) [7], the diagnostic performances of relative ROC curves were less accurate in predicting treatment response (Table 3).

**Table 3:** Comparison between diagnostic accuracy of different sTg thresholds for predicting treatment response 1 year after RAIT

sTg threshold	Sensitivity (95% CI)	Specificity (95% CI)	Youden index J
≥ 2 ng/ml	83.7% (74.2–90.8)	32.7% (24.2–42.2)	0.17
≥ 10 ng/ml	68.6% (57.7–78.2)	70.8% (61.5–79)	0.40
≥ 27.2 ng/ml	65.1% (54.1–75.1)	86.7% (79.1–92.4)	0.52

*CI* confidence intervals, *sTg* stimulated thyroglobulin

### Last disease status

After a median follow-up of 133 ± 100 months (range 12–360 months), NED was reported in 240 cases (87%), while persistent disease was observed in the remaining 34 (13%). Except of one patients died after surgery, all the others included in the study were alive at the last control.

Overall, of the 34 patients with persistent disease at last follow-up, 9 had evidence of structural disease, 9 of biochemical disease, and the remaining 16 both biochemical and structural disease.

At univariate analysis, sTg (as continuous variable and dichotomized with 27.2 ng/ml), 1 year treatment response categories, RAI first activity administered, total amount or RAI administered, and total number of RAIT were significantly correlated with the last disease status (Table 4). At multivariate analysis, only sTg (as continuous and cutoff derived) and 1-year treatment response categories were significantly associated with the risk of persistent disease (*p* = 0.023, *p* = 0.029, and < 0.001, respectively).

**Table 4:** Univariate and multivariate analyses for predictor of NED

	NED *n* = 240	Persistent disease *n* = 34	Univariate analysis		Multivariate analysis	
			*p* value	HR (95% CI)	*p* value	HR (95% CI)
Follow-up time, mean ± SD (months)	135 ± 104	136 ± 120	0.842	0.816 (0.412–1.415)		
Gender F:M	165:75	24:10	0.718	0.876 (0.432–1.858)		
Age, mean ± SD	14.5 ± 2.8	13.1 ± 3.8	0.631	1.250 (0.807–2.020)		
Puberty	189 (79%)	21 (62%)	0.137	0.595 (0.280–1.264)		
Capsule invasion	113 (47%)	25 (71%)	0.422	0.897 (0.333–1.4019)		
Vascular invasion	72 (30%)	16 (47%)	0.640	1.204 (0.614–2.441)		
Thyroiditis	57 (24%)	7 (21%)	0.514	0.774 (0.358–1.671)		
Previous history of cancer	31 (9%)	3 (9%)	0.840	0.890 (0.286–2.763)		
Histotype (PTC:fPTC:aggPTC:FTC:HC)	134:53:32:17:3	18:6:9:1:0	0.340	1.450 (0.777–2.109)		
Tumor size, mean ± SD (mm)	21 ± 13	25 ± 16	0.710	0.864 (0.400–1.866)		
Multicentricity	89 (37%)	11 (32%)	0.299	0.649 (0.349–1.382)		
ATA class risk (low-intermediate:high)	201:39	23:11	0.363	1.399 (0.651–2.976)		
sTg, mean ± SD (ng/ml)*	55 ± 111	134 ± 187	0.012	2.830 (1.255–6.389)	0.023	1.095 (0.411–2.920)
sTg ≥ 27.2 ng/ml*	61 (25%)	20 (57%)	< 0.001	3.650 (1.757–7.581)	0.029	1.122 (0.400–2.928)
sTg ≥ 10 ng/ml*	89 (37%)	23 (65%)	0.001	3.412 (1.585–7.344)	0.099	2.833 (0.807–10.056)
TgAb positive at ablation	62 (26%)	11 (31%)	0.196	1.870 (0.820–4.271)		
Presence of metastasis	31 (13%)	9 (26%)	0.545	0.777 (0.393–1.953)		
RAI first activity, mean ± SD (GBq)	2.6 ± 1.2	3.7 ± 2.9	0.001	0.310 (0.149–0.643)	0.149	2.2225 (1.109–5.601)
Total RAI, mean ± SD (GBq)	7.5 ± 12.3	12.5 ± 20.9	< 0.001	4.424 (2.215–9.018)	0.109	3.343 (0.785–15.593)
Total n° RAI therapies	1.8 ± 1.6	3 ± 3.9	0.002	2.882 (1.440–5.771)	0.258	0.322 (0.045–2.267)
1-year response (excellent: not excellent)	144:96	2:32	< 0.001	4.400 (2.214–8.821)	< 0.001	10.670 (1.716–66.454)

*NED* no evidence of disease, *HR* hazard ratio, *CI* confidence interval, *SD* standard deviation, *sTg* stimulated thyroglobulin, *Ab* antibodies, *RAI* radioactive iodine, *PTC* papillary thyroid carcinoma, *fPTC* follicular variant of papillary thyroid carcinoma, *aggPTC* aggressive variants of PTC, FTC/follicular thyroid carcinoma, *HC* Hurtle cell carcinoma

^*^In the multivariate analysis, they were evaluated separately to avoid multicollinearity

**Fig. 3:**
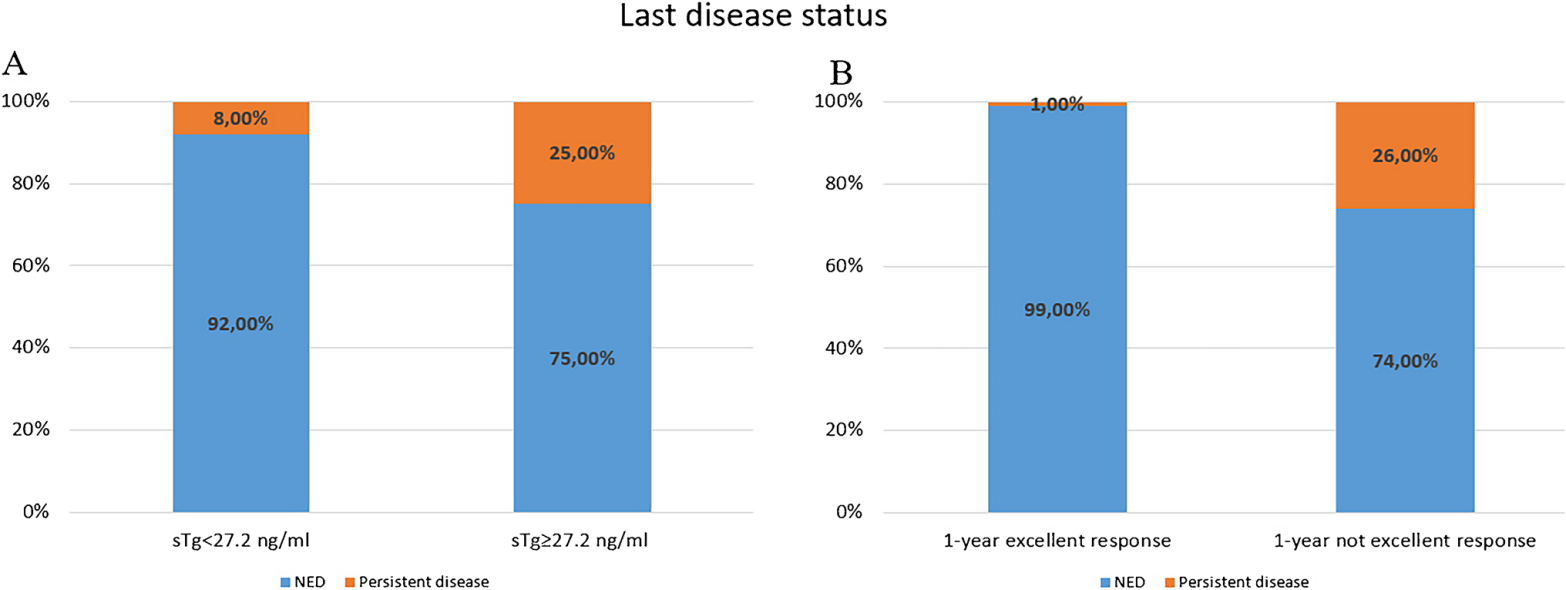
Evaluation of last disease status in patients with high or low sTg (**A**) and with excellent or not excellent response 1 year after RAIT (**B**)

Particularly, 25% of patients with baseline sTg ≥ 27.2 ng/ml showed structural o biochemical disease, while only 8% of patients with sTg < 27.2 ng/ml showed disease persistence at the end of follow-up (*p* < 0.001). Moreover, among patients with excellent response 1 year after the first RAIT, only 1% showed disease persistence, while among patients with excellent response, 26% showed structural or biochemical disease at the end of follow-up (*p* < 0.001) (Fig. 3).

Among 146 pediatrics with excellent response 1 year after RAIT, only two had structural recurrences (appearance of lung metastases in both cases) 2 years and 6 years after the diagnosis. They underwent four and five radiometabolic therapies for a total of 21.9 and 23.6 GBq of RAI, respectively, and showed persistent disease at the last follow-up.

Instead, among patients with 1-year incomplete response, a new surgery was performed in 6 cases, a second RAIT in 26 patients, and more than 2 RAITs in 60 cases. Finally 62 (68%) were NED, and the remaining 29 (32%) had persistent disease.

**Fig. 4:**
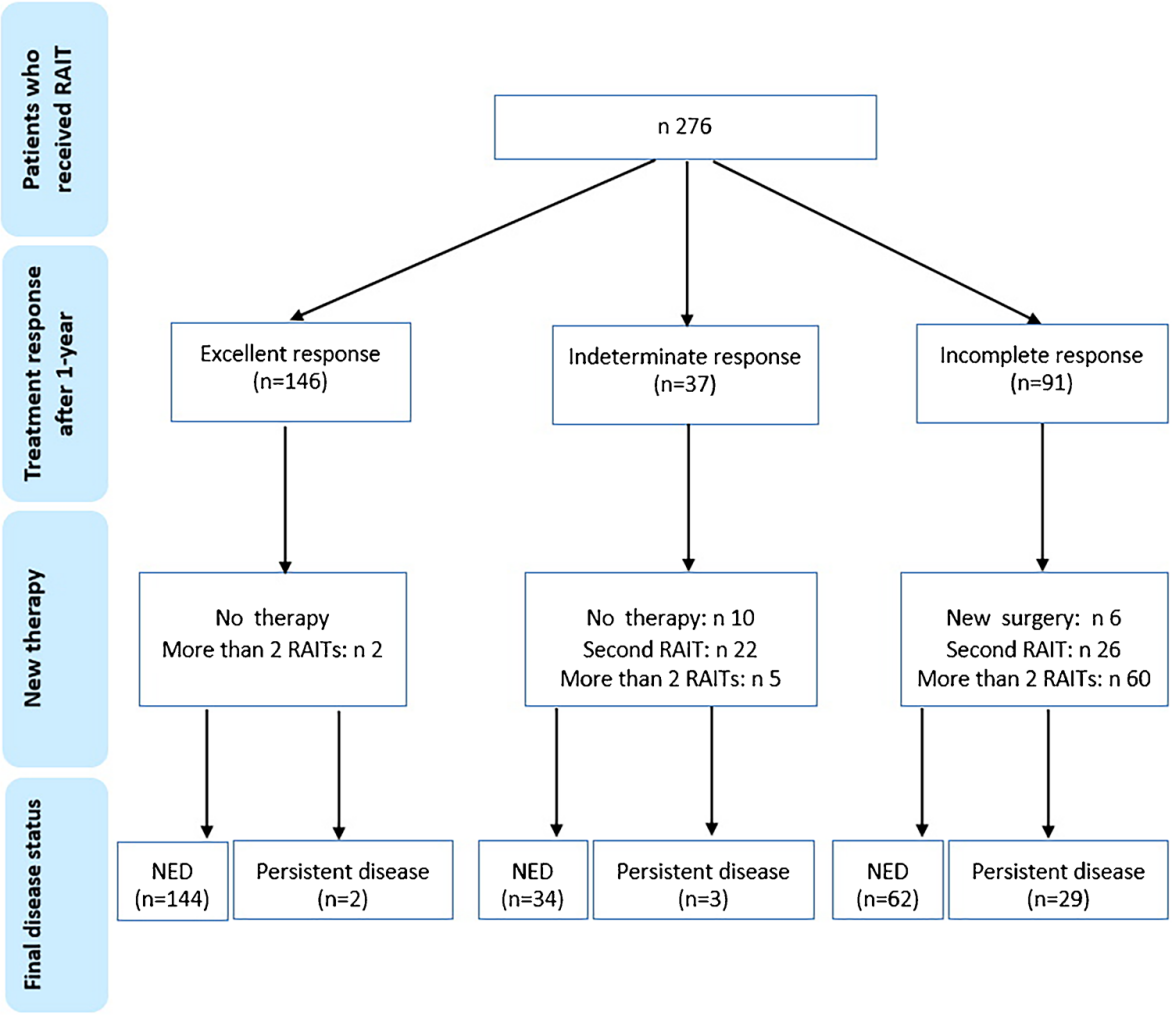
Flowchart of patients management after first RAIT

In the indeterminate response group (*n* = 37), only 10 patients followed a conservative protocol without receiving new therapies, while the other 27 underwent a new RAI treatment (in most cases, 22, only another radiometabolic therapy and in the remaining 5 more than 2 treatments) (Fig. 4).

## Discussion

In this multicentric study, we collected data from six different Italian centers widely distributed from Northern to Southern Italy. Despite the relative long period included (1990–2020) and the different geographical distribution, all center followed the same international guidelines to manage DTC patients, allowing to share these analyses.

The main aim of this study was to investigate the reliability of epidemiological, clinical, pathological, biochemical, and RAI-related variables to predict disease persistence/relapse 1 year after initial treatment (i.e., thyroidectomy + RAIT) and long-term survival in pediatric patients affected by DTC.

As the main finding, we demonstrated that sTg can predict excellent response 1 year after the first RAIT with the best cutoff value of 27.2 ng/ml. In this context, sTg seems to be the principal predictive value of disease response with a significantly higher impact on risk assessment when compared with the pediatric 2015 ATA guidelines risk classification. Moreover, we found that high levels of sTg and lack of excellent response 1 year after RAIT are parameters significantly associated to final outcome.

A risk-stratification system for recurrence/persistence of disease and a treatment response categories system after RAIT are the milestone of the recent ATA guidelines [3, 7] and are essential for providing the best care available for DTC patients.

DTC is usually a disease with good prognosis and optimal therapy response but may be associated with an excess of follow-up procedures, such as unnecessary surveillance, diagnostic tests, and medical appointments and even with overtreatment. This issue may have even more impact in pediatric patients, since these patients are expected to have lifelong follow-up and higher risk of RAIT-related side effects.

On the other hand, the early detection of those pediatric patients with aggressive disease and poor prognosis goes in the same direction as the purpose of a personalized therapy and management in the light of precision medicine, maximizing the efficacy and the necessity of RAIT.

In this context, the dynamic risk stratification and the individualization of treatment response categories proposed by the 2015 adults’ guidelines are particularly helpful but never applied in the pediatric context [3]. However, these classification systems are validated and shared mainly in adults [3] and not yet for pediatric population.

One of the major biases of pediatric DTC guidelines is the paucity of specific data and studies available, despite the fact that the peculiarity of the presentation, prognosis, and management of DTC in children has been clarified [4–6]. Thus, extrapolating and applying results from studies of adult population in pediatric field might lead to equivocal conclusions.

It is well known that total thyroidectomy and RAIT had a positive impact on disease free survival in pediatric patients [25–28], but the indication to RAIT is nowadays under debate especially in low-risk patients [7].

Awareness of the complications of treatment assumes increasing importance, making it imperative to balance the risks of treatment against potential gains from aggressive therapies; moreover, it makes mandatory a discussion of these potential risks with the patient and their parents throughout the course of their treatments.

Before us, only few studies have investigated the role of dynamic risk stratification in pediatrics with controversial results [12, 17, 20]. First, Lazar et al. [20] demonstrated that patients with excellent response had a better final prognosis when compared with not excellent response; even, all patients with incomplete response remained with persistent disease at the last control. Sung et al. [17] showed similar evidences reporting a higher risk of recurrent/persistent disease in the indeterminate and incomplete response group compared to excellent response. Indeed, our findings are in partial agreement with these papers [17, 20]. In a recent paper [29], response to therapy together with age and ATA risk predicted significantly event-free survival.

We found that patients with excellent and indeterminate response to initial treatment had similar good prognosis and a significant number of patients with incomplete response who were NED at the last follow-up.

Our results are concordant with those of Zanella et al. [12], showing in a relative small sample who underwent RAIT that only the dynamic risk stratification was independently associated with the last disease status (i.e., only univariate statistical model has been evaluated).

Beyond the importance of the 1-year treatment response classification, we demonstrated that sTg is associated with the final outcome and may predict response to RAIT. Tg is a specific and sensitive protein used as marker for the presence of follicular thyroid cells, and serum Tg measurement is a cornerstone tool in the management of DTC patients and is reasoned as the most sensitive method to detect persistent or recurrent disease. Few studies demonstrated the usefulness of sTg as prognostic factor in small and heterogeneous DTC pediatric populations and/or mixed with adults [12, 14, 16, 21, 30] and suggested various sTg cutoff values ranging from 10 to 37.8 ng/ml. This wide interval is directly related to the patients features recruited. For example, Klain et al. [14] including 45 patients with low to intermediate risk DTC demonstrated that a sTg of 10 ng/ml had the best accuracy to predict persistence of disease (sensitivity of 81%, specificity of 100%). On the contrary, Zanella et al. [12] proposed as reliable cutoff a sTg of 37.8 ng/ml having a sensitivity of 81% and a specificity of 100%. In our study, we got a threshold of 27.2 ng/ml to predict excellent response 1 year after RAIT and associated with disease outcome. This value seems to be more related to the clinical practice being derived from a larger sample and from different centers.

To validate the usefulness of this value, we compared it with other cutoff values proposed in literature such as 2 and 10 ng/ml [7]. Particularly, 10 ng/ml is a threshold often recurring and suggested also by Francis et al. [7], despite this value is not directly derived by pediatric population. Moreover, according to a meta-analysis [8], the best cutoff of sTg for predicting persistent disease in adult DTC patients is 10 ng/ml. The threshold proposed by us (27.2 ng/ml) is relatively higher than that proposed for adults, probably associated to the higher tumor burden of disease usually present at diagnosis in children, the presence of more frequently well-differentiated thyroid cells (a sort of more iodine-avidity cells), and the consequent best response to RAIT. However, the treatment response is optimal with an excellent response rate after 1 year registered in more than 50% and complete remission observed at the last control in 87%. Furthermore, a direct comparison between 10 and 27.2 ng/ml in the evaluation of the last disease status demonstrated a better performance of 27.2 ng/ml.

These observations might help to guide the follow-up of young patients with DTC, differentiating those who require a less intensive treatment from those who are candidates for more aggressive therapies and intense follow-up. However, it is fundamental to underline that all our patients received RAIT, and it could be reasonable to believe that the optimal response to therapy and good prognosis achieved is related to RAIT. In this context, analyzing our data is not possible to propose to avoid RAI in patients with low sTg at ablation.

Instead, ATA class risk stratification does not seem to have a predictive role in our analysis, and this evidence is similar to others [12, 20]. Thus despite the initial risk, the patients that received RAIT had a good response and optimal prognosis.

Our study contains several limitations, including its retrospective design, the potential use of heterogeneous management approaches over a relatively long period included, and the heterogeneity of laboratories methods applied related to each institutional protocol.

On the other hand, we must stigmatize the impossibility of having a homogeneity of approaches, methodologies, and tools considering the long period of 30 years in which the analysis has been retrospectively carried out. Despite the evident and significant methodological limitations, which are unavoidable, we still consider the results obtained from a real-life clinical practice in such a delicate sector as the pediatric one to be interesting and possibly useful. Further studies are desirable, but the data obtained can be considered a new and possibly useful piece in the mosaic of the clinical scenario of DTC in the pediatric field.

In conclusion, in pediatric DTC, a pre-ablation sTg ≥ 27.2 ng/ml is significantly associated with 1-year treatment response and with the risk of long-term persistent disease and therefore should be considered the principal factor that enables to identify patients who may need more intensive surveillance. Besides, 1-year response categories may also serve to predict the disease status at the last control.

## Supplementary Information

Below is the link to the electronic supplementary material.

Supplementary file1 (JPG 77 kb)
