# Supplementary figures and images for: Prognostic factors in children and adolescents with differentiated thyroid carcinoma treated with total thyroidectomy and RAI: a real-life multicentric study
Source: Eur J Nucl Med Mol Imaging. 2021 Oct 19;49(4):1374–85. doi: 10.1007/s00259-021-05586-8 (PMC8921094; doi:10.1007/s00259-021-05586-8)

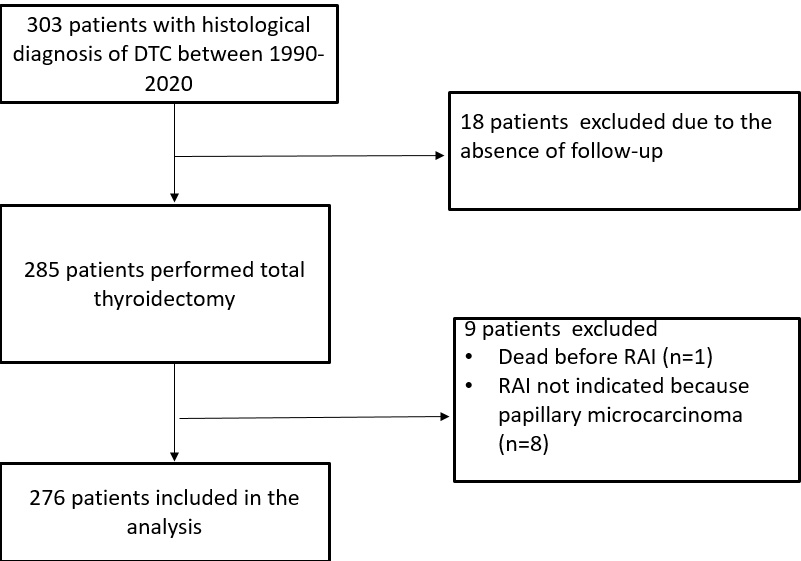

Supplement: Supplementary file 1 — Supplementary file1 (JPG 77 kb) [file 259_2021_5586_MOESM1_ESM.jpg]
